# Evaluating the Reliability of Three Different Dental Age Estimation Methods in Visakhapatnam Children

**DOI:** 10.5005/jp-journals-10005-1262

**Published:** 2015-02-09

**Authors:** Arun Kumar Patnana, Raja Sekhar Vabbalareddy, Narasimha Rao V Vanga

**Affiliations:** Postgraduate Student, Department of Pedodontics and Preventive Dentistry GITAM Dental College and Hospital, Visakhapatnam, Andhra Pradesh, India; Reader, Department of Pedodontics and Preventive Dentistry GITAM Dental College and Hospital, Visakhapatnam, Andhra Pradesh, India; Professor, Department of Pedodontics and Preventive Dentistry GITAM Dental College and Hospital, Visakhapatnam, Andhra Pradesh, India

**Keywords:** Age estimation, Chronologic age, Dental age, Demirjian's method, Willems method and Haavikko's method.

## Abstract

Dental age is important for treatment planning in the specialities of pedodontics and orthodontics. Although, Demirjian's method was considered standard for dental age estimation, it may not be reliable for all population.

**Aim:** The goal of the study was to evaluate the reliability of Demir-jian's, Haavikko's and Willems method of dental age estimation methods in Visakhapatnam (Andhra Pradesh, India) children.

**Study design:** One hundred and two children of 6 to 14 years old who underwent panaromic digital radiography for routine diagnostic purposes were included. Dental age was calculated using Demirjian's, Haavikko's and Willems methods and compared with chronologic age for each patient.

**Results:** Dental age showed a significant overestimation by Demirjian's method with a mean difference of 0.55 year and underestimation by Haavikko's and Willems methods with a mean difference of 1.95 and 0.20 year respectively when compared with chronologic age. The mean difference between dental age and chronologic age was not significant in Willems method which shows a close relation between dental and chronologic ages.

**Conclusion:** The dental age estimation by Willems method is found to be more accurate than Demirjian's and Haavikko's methods in Visakhapatnam children.

**How to cite this article:** Patnana AK, Vabbalareddy RS, Vanga NRV. Evaluating the Reliability of Three Different Dental Age Estimation Methods in Visakhapatnam Children. Int J Clin Pediatr Dent 2014;7(3):186-191.

## INTRODUCTION

The overall somatic maturity of a subject defines their physiological age. This age can be evaluated through the degree of maturation of one or more functional systems, such as skeletal, dental, or tegmental. Physiological age determination of individuals using all available scientific methods is common part of forensic practice and it is important for every identification process, especially when information relating to the deceased is not available. It is also helpful to identify corpses of unknown identity.^1^

In living persons, age estimation is done to assess whether the child has attained the age of responsibility, such as employment and marriage when the birth certificate is not available. In forensic odontology, there is a need to estimate the chronological age, which is the actual age of the accused/victim. The method of age estimation should be as accurate as possible, so that it enables the investigator to arrive at an age which is close to the chronologic age.^2^

The concept of physiological age determination is based on the degree of maturation of different tissue systems. Several biological ages have been developed which include: skeletal age, morphological age, secondary sexual characters and dental age. These criteria can be applied individually or together to measure the degree of physiological maturity of a growing child.^3^

Morphologic age is effected by the gender, ethnicity and environmental conditions; secondary sexual characters are infuenced by malnutrition and hormonal imbalances; and psychologic maturity is infuenced by the socioeconomic status and abuse conditions. Though skeletal age estimation is advantageous over previous methods, it is also infuenced by the hormonal imbal-ances.^4^ Dental age estimation is more reliable than above as it is less variable and genetically controlled.^5^

Dental age is of particular interest to an orthodontist for planning the treatment in different types of malocclusions. In pediatric endocrinopathies, the diagnosis and result of particular treatment may sometimes be better evaluated if dental age is assessed in parallel with other maturity indicators.^3^

Two different concepts of dental age estimation are present which include: (1) assessing eruption of teeth; (2) observing the mineralization of crowns and roots on radiographs of deciduous and permanent teeth.^6^

For many decades, tooth eruption is considered as standard dental age estimation method but now considered imprecise, because tooth eruption is infuenced by ankylosis, early or delayed extraction of primary teeth, impaction and crowding of permanent teeth.^3^ Alternatively, development of teeth using radiographs can be evaluated over long periods of time, in a continuous pattern using different stages of tooth formation as criteria.^7^

Among many proposed methods, Demirjian's (1973) system of age assessment is widely accepted. The advantage of the Demirjian's method includes the objective criteria describing stages of tooth development, which have been illustrated with line diagrams and radiographs very clearly.^3^ Demirjian's method is related on evaluation of one from eight appropriate radiographic stages (A to H) of crown and root development on permanent teeth from left side of mandible excluding 3rd molar. Demirjian's dental age estimation method was done in French-Canadian population but few studies showing that French Canadian standards were not applicable for age estimation in different population.^8-12^

As Demirjian's method is showed a significant over-estimation in other population, Willems (2001)^12^ modified the Demirjian's technique by creating new tables from which a maturity score could be directly expressed in years. The tiresome step of converting maturity score to dental age was omitted making it simpler, yet retaining the advantages of Demirjian's technique.

Another method using developing stages of both maxillary and mandibular teeth was introduced by Haavikko (1970).^13^ In this method, age estimation was based on determination of one of 12 radiographic stages (six relating to crown formation and six relating to root formation, with Stage ‘O’ allocated for appearance of a crypt of a tooth) of maxillary and mandibular teeth. It is based on a modified version of the tooth formation stages devised by Hunt and Gleiser (1955).^14^

Although various age assessment methods gave high degrees of reliability, ethnic differences between various population groups were found to affect the accuracy resulting in overestimation or underestimation of the dental age. Since the various studies for assessing the dental age have been conducted predominantly on the Western population, a similar assessment has been found to be lacking for Indian children. So, the aim of the present study was to evaluate the applicability of Demirjian's, Willems and Haavikko's methods of dental age estimation in Visakhapatnam (Andhra Pradesh, India) children.

## MATERIALS AND METHODS

A total of 102 orthopantomograms (OPGs) of children from 6 to 14 years age group were collected from the Department of Pedodontics and Preventive Dentistry, GITAM Dental College and Hospital. Personal data including date of birth, date of radiograph and gender were only collected from patients. The OPGs obtained were coded by a non-investigator in order to avoid bias during scoring of the radiographs. The investigator did not know the chronologic age of the children when assessing the radiographs. The patients with systemic diseases and congenital anomalies, premature birth, hypodontia of permanent teeth except 3rd molars were not included in the study.

In Demirjian's method, mandibular left teeth excluding the 3rd molars were assessed. Each tooth having a stage was converted into a score using the conversion table given by Demirjian and Goldstein for boys or girls, as appropriate. The scores of all seven teeth were added together to give the total maturity score. Dental age is assigned based on the maturity score using reference tables given by Demirjian.^3^

Methodology for Willems method is similar to Demir-jian's method. Developmental tooth stages according to Demirjian's technique^3^ with corresponding age scores expressed directly in years for each of the seven left man-dibular teeth for boys and girls. All scores were summed up to give dental age in years directly using the reference tables given by Willems.^12^

In Haavikko's method both maxillary and mandibular teeth were scored using the stage of tooth development. All the scores were summed up, dental age is given by dividing the summed up scores by number of teeth examined.^13^

Chronologic age was determined by subtracting the date of birth from the date of OPG. Dental age of each child was determined by Demirjian's, Willems and Haa-vikko's methods.

## STATISTICAL ANALYSIS

The data were statistically analyzed, mean difference between the dental age and chronologic age was calculated for each method. Paired t-test was done to evaluate the significance of mean difference between the three methods. Difference between the chronological ages of girls and boys was tested using independent samples t-test. Accuracy of each method was determined by mean difference between dental age and chronological age (dental age-chronological age) for girls and boys. The positive difference between dental age and chronologic age showed an overestimation while the negative difference refected an underestimation of dental age by the particular method. When the p-value was less than 0.05, the results were considered statistically significant.

**Table 1:** **Table 1:** Comparison of mean dental age estimated by Haavikko's, Demirjian's and Willems methods with chronologic age

		*Descriptive statistics*													
		*N*		*Range*		*Minimum in years*		*Maximum in years*		*Mean in years*		*Std. deviation*	
Chronological age		102		9.00		6.30		15.30		12.27		1.67	
Haavikko's method		102		7.10		6.80		13.90		10.31		1.04	
Demirjian's method		102		8.80		7.10		15.90		12.81		1.81	
Willems method		102		11.60		3.90		15.50		12.06		1.80	

**Table 2:** **Table 2:** Comparison of mean difference between Haavikko's, Demirjian's and Willems methods of dental age estimation with chronologic age

		*Mean*		*Std. deviation*		*Mean difference in years*		*Standard error*		*Paired t-test, p-value*		*Pearson's correlation coefficient*			
												*r*		*p-value*	
Chronological age		12.27		1.69		–1.95		0.12		0.001*		0.69		0.001*	
Haavikko's method		10.31		1.04											
Chronological age		12.27		1.69		0.55		0.08		0.001*		0.88		0.001*	
Demirjian's method		12.89		1.81											
Chronological age		12.27		1.67		–0.20		0.12		0.11		0.73		0.001*	
Willems method		12.06		1.80											

*Highly significant

**Graph 1:**
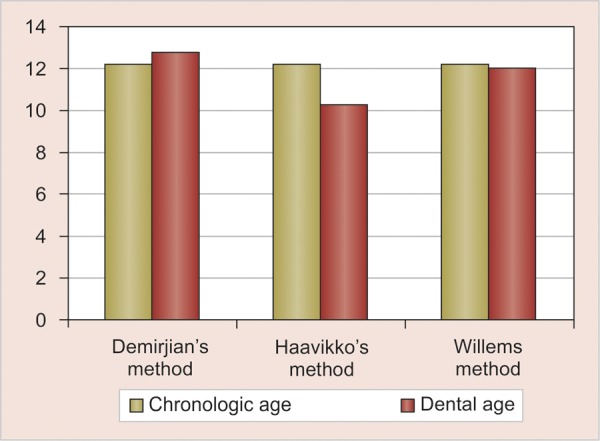
Comparison of dental age with chronologic age by Demirjian's, Haavikko's and Willems methods

**Graph 2:**
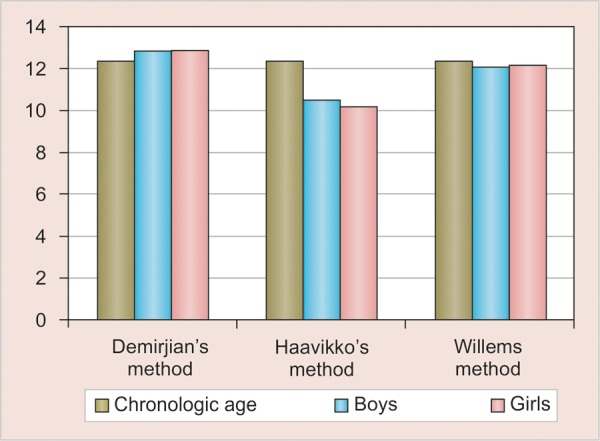
Gender-wise comparison of dental age with chronologic age between Demirjian's, Haavikko's and Willems methods

## RESULTS

The mean chronologic age for total 102 children was 12.2 years. The mean dental age by Haavikko's Demirjian's and Willems method were 10.31, 12.81 and 12.06 years respectively (Table 1). The mean difference between dental age and chronologic age for Haavikko's, Demirjian's and Willems method were –1.95, 0.55 and –0.20 years respectively (Table 2).

In the present study, Haavikko's and Willems method underestimated and Demirjian's method overestimated the dental age when compared to chronologic age. p-value showed no significant difference between chronologic age and dental age in the Willems method (p = 0.11). But, for Haavikko's and Demirjian's methods significant difference was found between chronologic age and dental age with a p-value of 0.001 (Graph 1).

In gender wise analysis for males, Willems method showed the least mean difference between chronologic age and dental age, i.e. 0.25 year. The mean difference between Haavikko's and Demirjian's method are 1.78 and 0.53 year respectively. For girls, the mean difference between dental age and chronologic age in Willems method is 0.15 year; by Haavikko's and Demirjian's methods, the mean difference was 0.19 and 0.14 year respectively. Paired t-test showed no significant difference was found between chronologic age and dental age in Willems method for both the boys and girls (Table 3 and Graph 2).

## DISCUSSION

Saunders, a dentist, was the first to publish information regarding dental implications in age assessment by presenting a pamphlet entitled ‘Teeth A Test of Age’ to the English parliament in 1837. While quoting the results from his study on 1000 children, he pointed out the value of dentition in age estimation.^15^

Developing teeth are used most reliably in age estimation. Teeth are the most indestructible part of the body and exhibit the least turnover of natural structure. They therefore not only survive death but also remain relatively unchanged thereafter for many thousands of years.^16^ The anticipated developmental sequence that human dentition follows, to reach complete dental development can be utilized in age determination.^17^

Several authors investigated the association between tooth emergence and root formation.^18^ Liliquist and Lindberg (1971)^19^ investigated a scoring system for maturity and Fanning (1971) has initiated a multivariate analysis approach. With the previous reports, it was concluded that tooth formation is more reliable indicator of dental maturity than gingival emergence or eruption.

The methods based on the stages of tooth formation seem to be more appropriate in the assessment of age as the dental development and calcification is controlled by genes rather than environmental conditions. Teeth are less susceptible to nutritional, hormonal and pathological changes.^20^

**Table 3:** **Table 3:** Gender-wise analysis of means between Haavikko's, Willems and Demirjian's methods of dental age estimation with chronologic age

				*Mean*		*Std. deviation*		*Mean difference*		*Standard error*		*Paired t-test p-value*		*Pearson's correlation coefficient*			
														*r*		*p-value*	
Males		Chronological age		12.27		1.70		–1.78		0.14		0.001*		0.857		0.001*	
		Haavikko's method		10.49		0.96											
		Chronological age		12.27		1.70		0.53		0.10		0.001*		0.917		0.001*	
		Demirjian's method		12.80		1.79											
		Chronological age		12.27		1.70		–0.25		0.22		0.25		0.617		0.001*	
		Willems method		12.01		1.91											
Females		Chronological age		12.26		1.69		–2.12		0.19		0.001*		0.574		0.001*	
		Haavikko's method		10.14		1.09											
		Chronological age		12.26		1.69		0.57		0.14		0.001*		0.840		0.001*	
		Demirjian's method		12.84		1.84											
		Chronological age		12.26		1.69		–0.15		0.12		0.22		0.862		0.001*	
		Willems method		12.11		1.71											

*Highly significant

In order to study dental formation, different developmental stages have been defined by several authors.^7,14,21^ These stages have usually been marked by recognizable tooth shapes, from the beginning of calcification through to fnal mature form on radiographs.

Radiology plays an indispensable role in human age determination as radiological images are routinely utilized in the process. Since 1982, dental radiography, a non-destructive and simple technique used daily in dental practice, has been employed in methods of age estimation.^22^

The radiographic dental age assessment methods are relatively simple and involve the identification of the stage of mineralization on radiographic images followed by their comparison with the standard stage to estimate the approximate age range. Various radiographic techniques like periapical, lateral oblique, cephalometric radiographs are recommended^20^ (however, panoramic radiographs are more commonly used than others).

Orthopantomographs are easy to make in young, nervous children than periapical radiographs. They give less radiation for a full mouth radiograph compared to periapical radiographs.^23^ Though, there was 3 to 10% of enlargement in OPGs it is not a drawback because the rating system is based on sharp criteria and relative values rather than on absolute lengths.^3^

Girls and boys were treated separately, because this allows for sex-tooth interaction that is for one tooth being relatively more advanced in one sex than in other. This is known to occur in tooth eruption and appears also in our scores, since they are higher for girls than boys in all teeth except 1st molar where girls are lower.^3^

The most widely used Demirjian's method is done in French-Canadian population. In this method, mandibular left teeth are considered for examination, if any tooth is missing then particular tooth from the contralateral side is examined for scoring. This method gives the similar results when compared with the 14 teeth (7 from mandibular left and 7 from mandibular right) examination method.^3^

Many studies have proved that Demirjian's method tends to overestimate the dental age both in boys and girls.^8-11^ Hence, Demirjian's method needs an adaptation for every specific population.^24-26^ However, a well-defined and reproducible stage of dental development of seven mandibular teeth and great number of different population on which the method was applied makes Demirjian's method suitable for age estimation.^27^

Serena Koshy and Shobha Tendon (1998) have done a study on the applicability of Demirjian's method in south Indian population in 184 children from 5 to 15 years of age and found an overestimation of dental age about 3.04 and 2.82 years in boys and girls respectively. Finally, they concluded that the assessment of dental age is dependent on the ethnic group on which it is to be tested and Demirjian's conversion of the maturity score to the dental age was not applicable for South Indian children.^8^

VM Phillips (2009) evaluated Moorrees, Fanning and Hunt (MFH) (1963) and Demirjian, Goldstein and Tanner (DGT) (1973) methods of dental age estimation in South African children and found that Moorrees method underestimates the age while Demirjian's method overestimates the dental age of these samples. Finally, they concluded that these methods are not applicable in South African juveniles and dental age related reference tables for the different ethnic groups in South Africa are necessary for age estimation of this children.^28^

Saifieddin Abu Asab (2011) evaluated the applicability of Demirjian's method in Kelantanese Malay children between 6 and 16 years of age and found that Demirjian's method overestimated the dental age by 1.23 years for boys and 1.20 years for girls and found that it was less accurate for the Kelantanese Malay children.^29^

In the present study population, Demirjian's method overestimated the dental age when compared to chronologic age significantly with a mean difference of 0.55 years. The overestimation of dental age in Demirjian's method might be because of two reasons, which include: (A) the study was done in French-Canadian population and so it may not be applicable to all the population, (B) dental maturation demonstrates few pubertal changes and thus is a poor indicator of pubertal growth spurt. In Demirjian's method, dental age is calculated from overall maturity score which may not be reliable. Dental maturation is rather independent from overall maturation in contrast to other maturational processes like skeletal or secondary sex character maturation, both of which are reported to be strongly correlated.^2^

Willems (2001) adapted a method for dental age estimation in a Belgian Caucasian population. It is a modifica-tion of Demirjian's method (New tables for boys and girls with age scores directly expressed in years). The adapted method was validated and resulted in more accurate dental age estimations in Belgian population.^12^ Willems, compared modified Demirjian tables with the original tables and found that dental age is overestimated by 0.5 to 0.6 years for boys and girls respectively in Demirjian's method and the overestimation is decreased to 0 to 0.2 years for boys and girls respectively in Willems method.

Shekhar Grover (2012) in Faridabad, India evaluated the accuracy of Willems methods and they found that an overestimation of dental age about 0.36 and 0.24 years in boys and girls respectively with chronologic age. The mean overestimation of age was less in girls than boys which was statistically significant.^30^

Vesna Ambarkova (2013), compared the accuracy of Demirjian's and Willems methods of dental age estimation methods in the Former Yugoslav Republic of Macedonia in 6 to 13 years old age group children and found that Willems method was the most accurate while Demirjian's methods for dental age calculation are not suitable on children in this population.^31^

Namratha Ramanan (2012) tested the applicability of Willems method of dental age estimation in 1877 Japanese children between 1 and 23 years age group and they found that the prediction tables developed by Willems (2001) in Belgian population could be used as a reference model to estimate the age of the Japanese individuals.^32^

In the present study population, Willems method showed an underestimation of age about 0.25 and 0.15 years in boys and girls which was not according to the other studies^2,30^ but, the correlation of this method with girls is more than boys which is coinciding with the previous studies Maber (2006)^33^ who found an overall underestimation of age using the Willems method in their population.

Andrea Carlo Butti (2009) tested the applicability of Haavikko's method of dental age estimation method in 500 healthy Caucasian children of 3.9 to 15.4 years age group and they found that there is a significant underestimation of dental age compared to chronologic age with Haavikko's method and they concluded that this method is not applicable in Italian population.^13^

In the present study population, dental age estimated by Haavikko's method is not applicable to Visakhapatnam children, because there is a significant underestimation of dental age by 1.78 and 2.12 years in boys and girls respectively, which was in accordance with the previous studies. Maber et al (2006)^33^ found Demirjian's method to be more accurate than Haavikko's method in their British and Bangladeshi samples.^26,33^

## CONCLUSION

This study comes out with a significant overestimation of dental age by Demirjian's method and underestimation of dental age by Haavikko's method. Willems method is found to be more accurate than Demirjian's and Haavik-ko's methods of dental age estimation in Visakhapatnam children. From the present study, it can be implied that assessment of dental age is dependent on diverse ethnic groups. Further research is needed on large population groups in order to improve the reliability and reproduc-ibility of the results.
